# Validation of the Modified Shuttle Test to Predict Peak Oxygen Uptake in Youth Asthma Patients Under Regular Treatment

**DOI:** 10.3389/fphys.2018.00919

**Published:** 2018-07-24

**Authors:** Fernanda C. Lanza, Mariana M. Reimberg, Raphael Ritti-Dias, Rebeca S. Scalco, Gustavo F. Wandalsen, Dirceu Sole, Marco van Brussel, H. J. Hulzebos, Simone Dal Corso, Tim Takken

**Affiliations:** ^1^Graduate Program in Rehabilitation Sciences, Universidade Nove de Julho, São Paulo, Brazil; ^2^Pediatric Department, Federal University of São Paulo, São Paulo, Brazil; ^3^Child Development and Exercise Center, University Medical Center Utrecht, Utrecht, Netherlands

**Keywords:** shuttle test, asthma, oxygen uptake, prediction equation, validation

## Abstract

**Background:** Oxygen uptake (VO_2_) evaluations by cardiopulmonary exercise test is expensive and time-consuming. Estimating VO_2_ based on a field test would be an alternative.

**Objective:** To develop and validate an equation to predict VO_2peak_ based on the modified shuttle test (MST).

**Methods:** Cross sectional study, with 97 children and adolescents with asthma. Participants were divided in two groups: the equation group (EG), to construct the equation model of VO_2peak_, and the cross-validation group (VG). Each subject performed the MST twice using a portable gas analyzer. The peak VO_2peak_ during MST was used in the equation model. The patients’ height, weight, gender, and distance walked (DW) during MST were tested as independent variables.

**Results:** The final model [-0.457 + (gender × 0.139) + (weight × 0.025) + (DW × 0.002)] explained 87% of VO_2peak_ variation. The VO_2peak_ predicted was similar to VO_2peak_ measured by gas analyzer (1.9 ± 0.5 L/min and 2.0 ± 0.5 L/min, respectively) (*p* = 0.67), and presented significant ICC 0.91 (IC95% 0.77 to 0.96); *p* < 0.001. The Bland–Altman analysis showed low bias (-0.15 L/min) and limits of agreement (-0.65 to 0.35 L/min). There was no difference in DW between EG (760 ± 209 m) and VG (731 ± 180 m), *p* = 0.51.

**Conclusion:** The developed equation adequately predicts VO_2peak_ in pediatric patients with asthma.

## Introduction

Asthma is a prevalent chronic pulmonary disease in the childhood. It is usually characterized by chronic airway inflammation that results in a decreased aerobic capacity, and its prevalence is increasing in many countries (Global Initiative for Asthma [GINA], 2014).

Cardiorespiratory fitness is considered an important marker of health in youth, since high cardiorespiratory fitness in youth have shown to track do adult ages (Hallal et al., 2006). In addition, children with higher cardiorespiratory fitness present lower prevalence of cardiovascular risk factors including obesity and high blood pressure (Boddy et al., 2014; Burns et al., 2016). Therefore, assessment of cardiorespiratory fitness can provide valuable information regarding health status in pediatric populations.

The measurement of maximal peak oxygen uptake (VO_2max_) have been considered the gold standard for cardiorespiratory fitness assessment (American Thoracic Society, 2003). However, due to its cost and time to assessment, field tests, such as incremental shuttle-walk test, have been proposed to assess cardiorespiratory fitness in clinical settings (Singh et al., 2014). In patients with asthma, a previous study analyzed the validity of shuttle-run test (Ahmaidi et al., 1993), a progressive externally-paced field test, to predict VO_2peak_ in adolescents with asthma, and observed that the shuttle-run test has sufficient validity to assess oxygen uptake. However, the severer asthmatic patients could not perform the shuttle-run because there is a high speed increasing.

In this context, field tests that includes walking, such as shuttle-walk test (Singh et al., 1992) or modified shuttle-walk test (Bradley et al., 1999) (MST), have been proposed for patients with pulmonary diseases. The studies have observed significant correlations between the performance in incremental shuttle-walk test and VO_2peak_, in adults with various chronic conditions, including asthma (Noonan and Dean, 2000; Mayorga-Vega et al., 2015). However, whether similar relationships occur in children or adolescents with asthma is unknown. Thus, the aims of the current study were (1) to evaluate the relationship between MST and VO_2peak_ in children and adolescents with asthma, and (2) to develop and validate an equation to predict VO_2peak_ based on MST test.

## Materials and Methods

### Patients

Patients between 6 and 18 years of age were recruited in 2013 through 2015 from the Pediatric Department of the Federal University of São Paulo, Brazil. All patients had been diagnosed by a pediatric physician, according to the GINA steps (Global Initiative for Asthma [GINA], 2014), as having asthma for at least 6 months, and had been on a stable treatment regime for at least 3 months. Patients were excluded from this study when no regular medication regime was being followed, if any exacerbation had occurred in the last 4 weeks before inclusion, or if the patient was not able to perform any of the tests needed for this study.

Patients were divided into two groups: (1) the equation group (EG), which included patients who were studied to construct the equation model of VO_2peak_, and (2) a cross-validation group (VG), which included patients for preliminary validation of the equation model. Patients were included in the study after their legal guardians read, agreed to, and signed the informed consent form, and the patient had signed the informed consent if they were 12 years of age or older. The study was approved by the Associaçao Educacional Nove de Julho Ethical Committee, São Paulo, Brazil (#738192/2014).

### Study Design and Protocol

This is a cross sectional study. All outcome measurements needed for this study were taken on two different days. The outcome measurements that were assessed included an asthma control questionnaire, lung function spirometry (pre- and post-bronchodilator), the MST and the cardiopulmonary exercise test (CPET). The MST and CPET were performed on different days, in random order.

### Asthma Control Questionnaire

The asthma control test (ACT) is a simple questionnaire to assess asthma control based on patient views, used for patients over 12 years old. The children’s ACT (C-ACT) is used for children 4 through 11 years old (Liu et al., 2007). Each question is answered on a scale of 1 (worst) to 5 (best). The highest total score for ACT is 25, and for C-ACT is 27. Asthma is considered controlled when the score is 20 or over. A score of 16 to 19 is considered partially-controlled asthma, and 15 or under is uncontrolled asthma (Liu et al., 2007).

### Lung Function

Spirometry was performed with ULTIMA CPX equipment (MGC Diagnostics Corporation. Saint Paul, MN, United States). The technical procedure, acceptance criteria and reproducibility were according to ATS/ERS statement (Wanger et al., 2005). The patients repeated the test after use of a bronchodilator (salbutamol, 400 μg). The forced vital capacity (FVC), expiratory forced volume at the first second (FEV_1_), FEV_1_/FVC, and forced expiratory flow at 25 to 75% of FVC (FEF_25-75_) were expressed in liters and percentage of predict value (Polgar and Promadhat, 1971), and z-score based on the mean value.

### Modified Shuttle Test

The modified shuttle test (MST) was performed in a 10-m-long corridor according to the previous description (Bradley et al., 1999). It is an externally-paced test dictated by an audio signal. The test has 15 levels, in which the speed increases every minute, ranging from 1.8 to 10.2 km/h, and requires the patient to walk/run at the increasing speed. The test was halted if the patient was not able to reach the distance two consecutive times, if he/she needed to stop due to fatigue or breathlessness, or if SpO_2_ fell below 82% (Singh et al., 2014). Each patient performed the test twice on the same day, with a 30-min rest in between. Heart rate, measured with a Polar FT70^®^ monitor, and SpO_2_, measured with a handheld oximeter (920M, Philips^®^, United States) were continuously evaluated. The modified Borg scale was used to observe lower limb fatigue and dyspnea at the beginning and end of the test (Borg, 1982). The greatest DW for the two MST, expressed in meters and as a percentage of predicted value (Lanza et al., 2015), was used for analysis as an outcome.

### Cardiopulmonary Exercise Test

The CPET was performed to identify if MST was maximal test. The cycle ergometer (Corival^®^, LODE BV Medical Technology, Groningen, Netherlands) was used. After 2 min of unloaded cycling, the load was increased (10 to 20 watts/minute) (Godfrey et al., 1971), and the test was aimed to last between 8 and 12 min (American Thoracic Society, 2003). The heart rate and SpO_2_ were continuously recorded. Blood pressure was measured every 2 min during the test. Modified Borg dyspnea and Modified Borg lower limb were assessed at beginning and the end of the test. The primary outcome of CPET was the VO_2peak_.

All patients performed the MST and CPET while connected to a validated (Kautza et al., 2004) system for gas exchange analyses (VO_2000_, MedGraphics Corporation^®^, Saint Paul, MN, United States). The VO_2_, pulmonary dioxide production (VCO_2_), the relationship between them (RER: VCO_2_/VO_2_), and minute ventilation (VE) were continuously measured.

### Statistical Analysis

The Shapiro–Wilk test was used to determine the normality of the data. The variables were described by mean ± SD or median (IQ 25 to 75%), according to the normality of the data. The paired *t*-student test was used to compare the characteristics between CPET and MST. The unpaired Student’s *t*-test or Mann–Whitney *U*-test was used to compare the characteristics of CPET and MST between EG and VG groups. The Pearson’s coefficient of correlation was performed to study the correlation between VO_2peak_ and age, height, weight, and distance walked. Subsequently, the multiple backward linear regression analysis was performed to identify predictors of the dependent variable (VO_2peak_) measured during MST. Age, height, weight, sex, and distance walked during MST were tested as independent variables. To validate the equation, the measured VO_2peak_ of the VG patients was compared to the predicted VO_2peak_. The intraclass correlation coefficient (ICC) and its 95% confidence interval (CI) were calculated using an absolute agreement definition, to evaluate the relative reliability between VO_2peak_ measured and predicted. The agreement between VO_2peak_ measured and predicted was assessed by Bland–Altman analysis. A sample size of at least 15 patients per variable was used in the final equation model developed using linear regression. The *post hoc* power of the validation group was calculated.

SPSS version 20 (Chicago, IL, United States) statistical software was used in the study. A *p*-value less than 0.05 was considered statistically significant.

## Results

A total of 153 children and adolescents with asthma were invited to participate, of which 97 were included (34 did not sign the informed consent form, 17 had asthma exacerbation occurring in the past 4 weeks, and five failed to follow the test procedure). Assuming a ratio 2:1, a total of 68 patients were randomly included in the equation group (56% boys). Another 29 VG patients (61% boys) were evaluated to validate the equation. They also had normal lung function and a median GINA step of 3 (2 to 4), **Table 1**.

**Table 1:** Anthropometric characteristics and lung function of the studied groups.

Variables	Equation group *N* = 68	Validation group *N* = 29	*p*
Age (years)	10.5 ± 3.2	10.8 ± 3.1	0.51
Weight (kg)	42.7 ± 15.7	40.5 ± 15.8	0.70
Height (cm)	143.0 ± 17.2	142.4 ± 14.7	0.73
FVC, L (%)	2.6 ± 0.8 (104 ± 12)	2.4 ± 0.8 (100 ± 13	0.33
FVC z-score^∗^	0.0 (-0.8 to 0.5)	0.0 (-0.5 to 0.3)	0.78
FEV_1_, L (%)	2.2 ± 0.7 (100 ± 14)	2.0 ± 0.6 (93 ± 15)	0.12
FEV_1_ z-score^∗^	-0.1 (-0.6 to 0.6)	-0.1 (-0.7 to 0.8)	0.93
FEV_1_/FVC	88 ± 6	85 ± 9	0.33
FEV_1_/FVC z-score^∗^	0.2 (-0.6 to 0.7)	0.2 (-0.8 to 0.7)	0.95
FEF_25-75_, L (%)	2.9 ± 1.1 (114 ± 35)	2.4 ± 1.0 (103 ± 35)	0.22
FEF_25-75_ z-score^∗^	-0.1 (-0.8 to 0.8)	-0.1 (-0.7 to 0.3)	0.72
Asthma control test^∗^	21 (18 to 23)	20 (18 to 22)	0.44


*FVC, forced vital capacity; FEV_*1*_, forced expiratory volume at 1^*st*^ second of FVC; FEF, flow expiratory volume at 25–75% of FVC; ^∗^median (IQ 25–75%).*

Of 84 (86%) of 97 patients performed the CPET according to the recommendations. The variables of CPET and MST were compared to be sure that a field test (the MST) had similar responses to the gold standard test. They had a significantly higher VO_2peak_ at MST (1.9 ± 0.6 L/min) than they did at CPET (1.5 ± 0.4 L/min), *p* < 0.001. A similar result was observed for their heart rate (MST: 186 ± 13 bpm vs. CPET: 178 ± 11 bpm; *p* < 0.001), but not statically difference for RER_peak_ between tests (MST: 1.1 ± 0.1 vs. CPET: 1.1 ± 0.1; *p* = 0.29).

**FIGURE 1:**
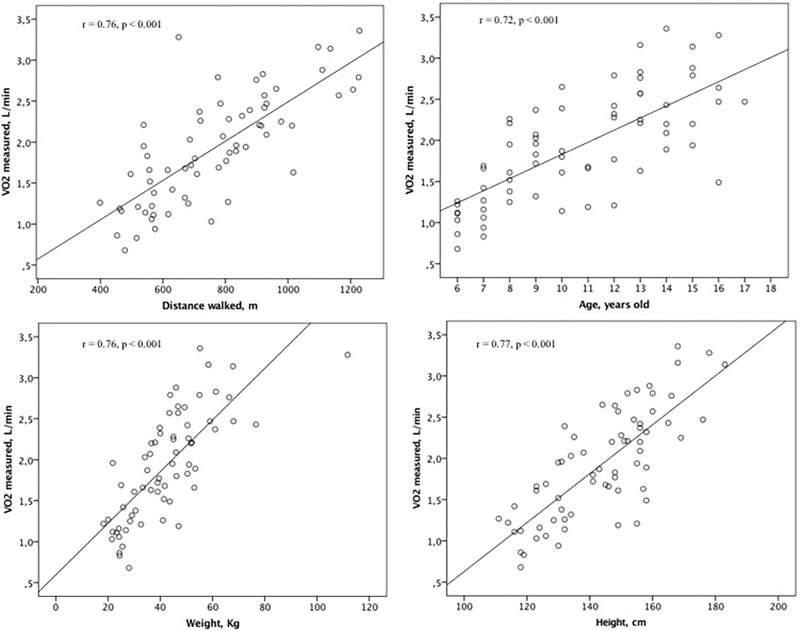
Association between VO_2peak_, distance walked, age, weight, and height for the equation group.

All patients stopped the MST at maximal effort (leg fatigue or breathlessness reported on the Borg scale, or incapacity to follow the test speed). There was a significant association between VO_2peak_, distance walked, age, weight, and height (*r* > 0.7; *p* < 0.05) for the EG (**Figure 1**). The final model of the linear multiple regression analysis included gender, weight, and distance walked during MST. The standardized coefficient β is shown in **Table 2**.

**Table 2:** Predicted variables for peak oxygen uptake, VO_2peak_ in L/min, based on the modified shuttle test obtained from multiple linear regression analysis.

Variables	Unstandardized coefficients β	Standardized coefficients β	*p*
Constant	-0.457		
Gender	0.139	0.103	<0.001
Weight (kg)	0.025	0.585	<0.001
Distance walked (m)	0.002	0.527	<0.001


*Gender: boys = 1; girls = 0.*

EquationVO2peak(Lmin)=−0.457+(gender×00.139)+(weight×00.025)+(DW×00.002)

Adjusted R^2^: 0.87; SEE: 0.24;
Gender: boys = 1; girls = 0;
Weight: kg;
DW: distance walked at MST, in meters.

**FIGURE 2:**
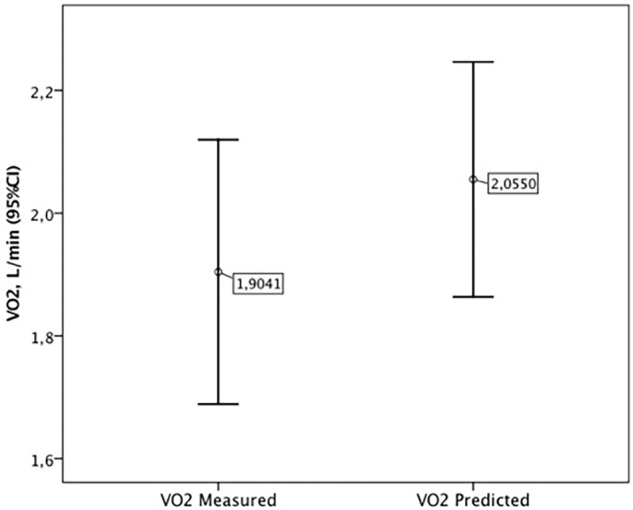
Comparison between VO_2peak_ measured and VO_2peak_ predicted, *p* = 0.67.

There was no difference between the equation and validations groups in the variables at the peak of MST (**Table 3**). No adverse events were reported during or after the MST. The VO_2peak_ measured during the MST of the validation group (1.9 ± 0.5 L/min) was not different of the VO_2peak_ predicted by the equation (2.0 ± 0.5 L/min), *p* = 0.67 (**Figure 2**). The *post hoc* power between the VO_2peak_ measured and predicted was 83%.

**Table 3:** Variables at the peak of the modified shuttle test in both groups.

Variables	Equation group *N* = 68	Validation group *N* = 29	*p*
DW, m (%pred)	760 ± 209 (80 ± 14)	731 ± 180 (77 ± 15)	0.51
HR_peak_, bpm (%pred)	185 ± 14 (92 ± 8)	187 ± 11 (93 ± 6)	0.51
VO_2peak_, L/min	1.9 ± 0.6	1.9 ± 0.5	0.92
VCO_2peak_, L/min	2.0 ± 0.8	2.0 ± 0.7	0.94
RER_peak_	1.0 ± 0.1	1.0 ± 0.1	0.86
VE_peak_, L/min	45.4 ± 14.5	47.0 ± 16.4	0.66
VE/VO_2peak_	23.6 ± 3.0	23.8 ± 2.2	0.82
VE/VCO_2peak_	22.9 ± 3.2	23.2 ± 2.8	0.73
SpO_2peak_, %	90 ± 5	91 ± 5	0.60
Desaturation, %^∗^	-6 (-10 to -2)	-5 (-10 to -2)	0.98
Borg dyspnea^∗^	4 (3 to 5.5)	3 (3 to 5)	0.57
Borg lower limb^∗^	4 (3 to 6)	3.5 (2 to 6)	0.67


*DW, distance walked at MST; HR, heart rate; VO_*2peak*_, peak oxygen uptake; VCO_*2peak*_, carbon dioxide production; RER, respiratory exchange ratio; VE, minute ventilation; ^∗^median (IQ 25–75%).*

The ICC of the VO_2peak_ measured and predicted in the validation group was highly significant (ICC: 0.91, CI95% 0.77 to 0.96; *p* < 0.001). The Bland–Altman analysis showed a mean bias of -0.15 L/min, with the limits of agreement ranging from -0.65 to 0.35 L/min.

## Discussion

The main results of the present study were: (i) gender, weight, and DW during MST explained 87% of the variance in VO_2peak_; (ii) There was no difference between the equation and validations groups in the variables at the peak of MST; (iii) The ICC of the VO_2peak_ measured and predicted in the validation group was highly significant; (iv) MST elicited higher peak VO_2_ and heart rate than CPET.

It was observed highest VO_2peak_ and heart rate during MST compared to the CPET. This result occurred because the CPET was performed on a cycle ergometer and MST is a walk/run test. The CPET is considered a gold standard assessment for exercise capacity, regardless of whether it is performed on a treadmill or a cycle ergometer (American Thoracic Society, 2003), but it is known that VO_2peak_ is higher on a treadmill (Maffeis et al., 1994).

As expected weight and gender were included in the VO_2peak_ prediction equation. It is known that the absolute peak oxygen uptake is related to a child’s growth (Vinet et al., 2003). Usually, the VO_2peak_ is 20% higher in children than in adults (Williams et al., 1985). This is because children have higher capillary and mitochondrial density, and oxidative enzyme activity than do adults (Williams et al., 1985; Rowland et al., 2000; Armstrong and Barker, 2009). Gender also influences the VO_2peak_ (Rowland et al., 2000). Girls have usually lower VO_2peak_ levels compared to boys, at least for the onset of puberty (Armstrong and Welsman, 1994). Oxygen uptake is related to the stroke volume of the heart, which is greater in boys, justifying the gender differences (Willmore et al., 2008). Additionally, boys have a higher percentage of free-fat mass (especially after puberty) compared to girls, which further increases their oxygen uptake during maximal exercise (Rowland et al., 2000; Vinet et al., 2003).

In same way, the distance walked was included in the final model of VO_2peak_ equation. Because of the incremental increase of the walking speed every minute of MST, the distance walked is related to the intensity of the exercise, and hence, the oxygen uptake. This is in agreement with previous studies in adults (Léger et al., 1988; Larsen et al., 2002; Weisgerber et al., 2009; Ross et al., 2010) that also included the distance walked to predict VO_2peak_. However, in these studies this variable explained less than 50% of the VO_2peak_, while in the current study DW, gender and weight explained 87%. This can be justified because these independent variables are proportional related to the oxygen uptake.

This is the first study to determine an equation for prediction of VO_2peak_ based on the MST in children and adolescents with asthma. The results in the cross-validation asthmatic patients revealed a high correlation between the predicted and obtained VO_2peak_, with small bias between them -0.15 L/min. In addition, although there was a wide limit of agreement, greater than 0.5 L/min (∼25% of VO_2peak_), it is similar to the results of Ahmaidi et al. (1993) who observed similar variability (∼25% of VO_2peak_) between VO_2peak_ measured and predicted in a study of patients with adolescents with asthma.

In practical terms, our proposed equation to estimate VO_2peak_ in children and adolescents from 6 to 18 years with asthma presented psychometric characteristics similar than other equations in adolescent and adult patients with asthma. The MST seems to be an appropriate test to evaluate pediatric patients, because it is performed at a wide range of speeds, and this avoids floor or ceiling effects (Saglam et al., 2016).

The limitation of this study includes the small number of patients with severe asthma (GINA step 5), which reduced the external validity for the equation in this subgroup of patients. The CPET was not properly performed in all participants, but more than 80% of them performed it. There was not included a control group of healthy participants, and whether differences exist between healthy and asthmatic youth cannot be determined. However, considering that equations for this specific group was not available until now, the inclusion of only asthmatic still relevant to determine an equation for pulmonary chronic conditions.

## Conclusion

The developed equation indicated high correlation and low bias in the prediction VO_2peak_ based in the MST in pediatric patients with asthma. Therefore, this test can be a useful alternative to assess VO_2peak_ in clinical settings.

## Author Contributions

FL had substantial contributions to the study including: conceptualization/design; supervision/oversight; funding acquisition; formal analysis; drafting significant parts of the work. MR and RS had substantial contributions to the study including: methodology, investigation, data curation. RR-D, GW, and DS had substantial contributions to the study including: critically revising it so as to contribute to the interpretation. MvB, HH, SDC, and TT had substantial contributions to the study including: analysis and interpretation of research data drafting significant parts of the work or critically revising.

## Conflict of Interest Statement

The authors declare that the research was conducted in the absence of any commercial or financial relationships that could be construed as a potential conflict of interest.

## References

[B1] Ahmaidi S. B. Varray A. L. Savy-Pacaux A. M. Prefaut C. G. (1993). Cardiorespiratory fitness evaluation by the shuttle test in asthmatic subjects during aerobic training. *Chest* 103 1135–1141. 10.1378/chest.103.4.1135 8131453

[B2] American Thoracic Society (2003). American College of Chest Physicians. ATS/ACCP Statement on cardiopulmonary exercise testing. *Am. J. Respir. Crit. Care Med.* 167 211–277. 10.1164/rccm.167.2.211 12524257

[B3] Armstrong N. Barker A. R. (2009). Oxygen uptake kinetics in children and adolescents: a review. *Pediatr. Exerc. Sci.* 21 130–147. 10.1123/pes.21.2.130 19556620

[B4] Armstrong N. Welsman J. R. (1994). Assessemt and interpretation of aerobic fitness in children and adolescents. *Exerc. Sport Sci. Rev.* 22 435–476. 10.1249/00003677-199401000-00016 7925551

[B5] Boddy L. M. Murphy M. H. Cunningham C. Breslin G. Foweather L. Gobbi R. (2014). Physical activity, cardiorespiratory fitness, and clustered cardiometabolic risk in 10- to 12-year-old school children: the REACH Y6 study. *Am. J. Hum. Biol.* 26 446–451. 10.1002/ajhb.22537 24599609

[B6] Borg G. A. (1982). Psychophysical bases of perceived exertion. *Med. Sci. Sports Exerc.* 14 377–381. 10.1249/00005768-198205000-00012 7154893

[B7] Bradley J. Howard J. Wallace E. Elborn S. (1999). Validity of a modified shuttle test in adult cystic fibrosis. *Thorax* 54 437–439. 10.1136/thx.54.5.437 10212110 PMC1763768

[B8] Burns R. D. Brusseau T. A. Fang Y. Fu Y. Hannon J. C. (2016). Waist-to-Height ratio, aerobic fitness, and cardiometabolic risk in hispanic children from low-income U.S. Schools. *Pediatr. Exerc. Sci.* 28 388–396. 10.1123/pes.2016-0016 27174796

[B9] Global Initiative for Asthma [GINA] (2014). Available at: www.ginasthma.org

[B10] Godfrey S. Davies C. T. Wozniak E. (1971). Cardio-respiratory response to exercise in normal children. *Clin. Sci.* 40 419–431. 10.1042/cs0400419 5556096

[B11] Hallal P. C. Victora C. G. Azevedo M. R. Wells J. C. (2006). Adolescent physical activity and health: a systematic review. *Sports Med.* 36 1019–1030. 10.2165/00007256-200636120-00003 17123326

[B12] Kautza B. C. Kastello G. Sothmann M. S. (2004). Validation of MedGraphics’ VO2000 portable metabolic analyzer and a modified pneumotachometer. *Med. Sci. Sports Exerc.* 3:S222 10.1249/00005768-200405001-01063

[B13] Lanza F. C. Zagatto Edo P. Silva J. C. Selman J. P. Imperatori T. B. Zanatta D. J. (2015). Reference equation for the incremental shuttle walk test in children and adolescents. *J. Pediatr.* 167 1057–1061. 10.1016/j.jpeds.2015.07.068 26323195

[B14] Larsen G. E. George J. D. Alexander J. L. Fellingham G. W. Aldana S. G. Parcell A. C. (2002). Prediction of maximum oxygen consumption from walking, jogging, or running. *Res. Q. Exerc. Sport* 73 66–72. 10.1080/02701367.2002.10608993 11926486

[B15] Léger L. A. Mercier D. Gadoury C. Lambert J. (1988). The multistage 20 metre shuttle run test for aerobic fitness. *J. Sports Sci.* 6 93–101. 10.1080/02640418808729800 3184250

[B16] Liu A. H. Zeiger R. S. Sorkness C. A. Mahr T. Ostrom N. Burguess S. (2007). Development and cross-sectional validation of the childhood asthma control test. *J. Allergy Clin. Immunol.* 119 817–825. 10.1016/j.jaci.2006.12.662 17353040

[B17] Maffeis C. Schena F. Zaffanello M. Zoccante L. Schutz Y. Pinelli L. (1994). Maximal aerobic power during running and cycling in obese and non-obese children. *Acta Paediatr.* 83 113–116. 10.1111/j.1651-2227.1994.tb12965.x 8193460

[B18] Mayorga-Vega D. Aguilar-Soto P. Viciana J. (2015). Criterion-related validity of the 20-m shuttle run test for estimating cardiorespiratory fitness: a meta-analysis. *J. Sports Sci. Med.* 14 536–547. 26336340 PMC4541117

[B19] Noonan V. Dean E. (2000). Submaximal exercise testing: clinical application and interpretation. *Phys. Ther.* 80 782–807. 10911416

[B20] Polgar G. Promadhat V. (1971). *Pulmonary Function Testing in Children: Techniques and Standards.* Philadelphia, PA: Saunders.

[B21] Ross R. M. Murthy J. N. Wollak I. D. Jackson A. S. (2010). The six minute walk test accurately estimates mean peak oxygen uptake. *BMC Pulm. Med.* 10:31. 10.1186/1471-2466-10-31 20504351 PMC2882364

[B22] Rowland T. Goff D. Martel L. Ferrone L. (2000). Influence of cardiac functional capacity on gender differences in maximal oxygen uptake in children. *Chest* 117 629–635. 10.1378/chest.117.3.629 10712984

[B23] Saglam M. Vardar-Yagli N. Savci S. Inal-Ince D. Aribas Z. Bosnak-Guclu M. (2016). Six minute walk test versus incremental shuttle walk test in cystic fibrosis. *Pediatr. Int.* 58 887–893. 10.1111/ped.12919 26756566

[B24] Singh S. J. Morgan M. D. Scott S. Walters D. Hardman A. E. (1992). Development of a shuttle walking test of disability in patients with chronic airways obstruction. *Thorax* 47 1019–1024. 10.1136/thx.47.12.1019 1494764 PMC1021093

[B25] Singh S. J. Puhan M. A. Andrianopoulos V. Hernandes N. A. Mitchell K. E. Hill C. J. (2014). An official systematic review of the European Respiratory Society/American thoracic society: measurement properties of field walking tests in chronic respiratory disease. *Eur. Respir. J.* 44 1447–1478. 10.1183/09031936.00150414 25359356

[B26] Vinet A. Mandigout S. Nottin S. Nguyen L. Lecoq A. M. Courteix D. (2003). Influence of body composition, hemoglobin concentration, and cardiac size and function of gender differences in maximal oxygen uptake in prepubertal children. *Chest* 124 1494–1499. 10.1378/chest.124.4.1494 14555585

[B27] Wanger J. Clausen J. L. Coates A. Pedersen O. F. Brusasco V. Burgos F. (2005). ATS/ERS task force. Standardisation of the measurement of lung volumes. *Eur. Respir. J.* 26 511–522. 10.1183/09031936.05.00035005 16135736

[B28] Weisgerber M. Danduran M. Meurer J. Hartmann K. Berger S. Flores G. (2009). Evaluation of Cooper 12-minute walk/run test as a marker of cardiorespiratory fitness in young urban children with persistent asthma. *Clin. J. Sport Med.* 19 300–305. 10.1097/JSM.0b013e3181b2077a 19638824

[B29] Williams C. A. Carter H. Jones A. M. Doust J. H. (1985). Oxygen uptake kinetics during treadmill running in boys and men. *J. Appl. Physiol.* 90 1700–1706. 10.1152/jappl.2001.90.5.1700 11299258

[B30] Willmore J. H. Costill D. L. Kenney W. L. (2008). *Physiology of Sport and Exercise.* Champaign, IL: Human Kinetics, 24–45.

